# Evaluation of the Dermatoprotective Properties of *Clinopodium nepeta* and *Thymus vulgaris* Essential Oils: Phytochemical Analysis, Anti‐Elastase, Anti‐Tyrosinase, Photoprotective Activities, and Antimicrobial Potential Against Dermatopathogenic Strains

**DOI:** 10.1111/srt.70191

**Published:** 2025-06-13

**Authors:** Mohamed Taibi, Amine Elbouzidi, Nour Eddine Bentouhami, Mounir Haddou, Abdellah Baraich, Yousra Hammouti, Yousra Belbachir, Reda Bellaouchi, Ramzi A. Mothana, Mohammed F. Hawwal, Abdeslam Asehraou, Salwa Karboune, Mohamed Addi, Bouchra El Guerrouj, Khalid Chaabane

**Affiliations:** ^1^ Laboratory of Agricultural Production Improvement Biotechnology, and Environment (LAPABE) Faculty of Sciences Mohammed First University Oujda Morocco; ^2^ Laboratory of Bioresources Biotechnology Ethnopharmacology and Health Faculty of Sciences Mohammed First University Oujda Morocco; ^3^ Department of Chemistry Physical Chemistry of Natural Resources and Process Team Laboratory of AppliedChemistry and Environment (LCAE) Faculty of Sciences Mohammed First University Oujda Morocco; ^4^ Department of Pharmacognosy College of Pharmacy King Saud University Riyadh Saudi Arabia; ^5^ Department of Food Science and Agricultural Chemistry Macdonald Campus Quebec Canada

**Keywords:** anti‐elastase, antifungal, antibacterial, anti‐tyrosinase, *Clinopodium nepeta*, photoprotection, *Thymus vulgaris*

## Abstract

**Background:**

The growing demand on natural ingredients in cosmetics has immensely contributed to a renewed interest in cosmetic industry in plant derivatives, especially essential oils. The aim of this study is to examine the dermatoprotective and antifungal properties of *Clinopodium nepeta* (CNEO) and *Thymus vulgaris* (TVEO) essential oils.

**Materials and Methods:**

Gas chromatography‐mass spectrometry (GC‐MS) analysis was performed to identify the chemical composition of the essential oils. Anti‐elastase and anti‐tyrosinase activities were evaluated using standard enzymatic inhibition assays, and IC_50_ values were calculated. Photoprotective properties were determined using in vitro sun protection factor (SPF) calculations. Antifungal and antibacterial activities were assessed using the disc diffusion method and minimum inhibitory concentration (MIC) determination against *Candida albicans*, *Candida glabrata, Micrococcus luteus*, and *Staphylococcus aureus*.

**Results:**

GC‐MS analysis revealed the presence of 13 compounds in CNEO, mainly oxygenated monoterpenes (91.9%) with pulegone (42.3%) as the main component, and 25 compounds in TVEO, with α‐terpineol (19.8%) and carvacrol (13.5%) as the dominant compounds. CNEO showed superior anti‐elastase activity (IC_50_ = 13.55 ± 0.81 µg/mL) compared with TVEO (IC_50_ = 28.40 ± 2.64 µg/mL). Both oils demonstrated significant anti‐tyrosinase effects, with CNEO showing greater efficacy in inhibiting monophenolase (IC_50_ = 36.71 ± 4.09 µg/mL) and diphenolase (IC_50_ = 22.77 ± 0.97 µg/mL) than TVEO. SPF calculations revealed notable photoprotective properties for both oils, with CNEO (SPF = 6.472) slightly outperforming TVEO (SPF = 5.640). Antifungal tests against *C. albicans* and *C. glabrata*, and antibacterial tests against *M. luteus* and *S. aureus* showed that both oils possess strong antifungal and antibacterial activities, with CNEO demonstrating superior efficacy (MIC = 0.50 ± 0.00% v/v for both *Candida* strains) compared with TVEO (MIC = 0.011 ± 0.00% v/v for both *Candida* strains).

**Conclusion:**

This study provides the first comprehensive assessment of the dermatoprotective, antifungal, and antibacterial activities of CNEO and confirms the potential of TVEO in cosmetic, antifungal, and antibacterial applications. The results suggest that these essential oils could serve as promising natural ingredients in dermatoprotective, antifungal, and antibacterial formulations.

## 1 Introduction

Dermal diseases represent a major public health challenge, affecting millions of people worldwide [1]. These skin conditions can be caused by a variety of factors, including dysfunctional enzymes and dermatopathogenic germs [2, 3]. The most common dermal problems are acne, eczema, psoriasis, and fungal infections. Enzymes are crucial in maintaining skin homeostasis, but their dysregulation can lead to significant disorders [4]. Similarly, dermatopathogenic germs, such as certain bacteria and fungi, can colonize the skin and cause potentially serious infections [5, 6].

Elastase and tyrosinase are two enzymes of particular importance in the context of dermal diseases. When overactive, elastase can excessively degrade elastin, leading to loss of skin elasticity and premature aging [7]. Tyrosinase, for its part, is involved in melanin production, and its dysfunction can lead to pigmentation disorders [8]. In addition, pathogens such as *Candida albicans*, *Candida glabrata*, *Staphylococcus aureus*, and *Micrococcus luteus* are responsible for various skin infections, ranging from superficial mycoses to deeper bacterial infections [9, 10, 11, 12].

Aromatic plants have attracted growing interest in the field of dermatoprotection due to their wealth of bioactive molecules. These compounds, such as polyphenols (including flavonoids), terpenes, and other bioactive compounds, exhibit a variety of biological activities beneficial to the skin. Their antioxidant, anti‐inflammatory, and antimicrobial properties make them promising candidates for the development of natural treatments for skin disorders [13, 14, 15].

Essential oils, extracted from various aromatic plants, are recognized for their multiple biological activities. Their antimicrobial, anti‐inflammatory, and antioxidant properties make them particularly useful in the field of dermatology [16, 17]. Some essential oils have shown beneficial effects against acne, eczema, and other skin conditions, offering natural alternatives to conventional treatments [18, 19].

The Lamiaceae family includes many aromatic plants, among which *Clinopodium nepeta* (calament nepeta) and *Thymus vulgaris* (common thyme) were specifically selected for this study based on two main factors [20, 21]. First, previous studies have highlighted the significant biological activities of these essential oils, particularly their antimicrobial and antioxidant properties. Second, their widespread availability in the Eastern region of Morocco made them accessible candidates for this research. Both species are renowned for their essential oils, rich in bioactive compounds. The essential oil of *C. nepeta* is characterized by its high content of pulegone and menthone, while that of *T. vulgaris* is rich in thymol and carvacrol [22, 23]. These essential oils have demonstrated significant anti‐inflammatory and antimicrobial activities, making them promising for dermatological applications [24, 25].

This study aims to carry out a phytochemical characterization and evaluate the dermatoprotective activities of *C. nepeta* and *T. vulgaris* essential oils. We will focus on their inhibitory potential against two enzymes with adverse effects on the skin: tyrosinase and elastase. In addition, we will evaluate their antimicrobial efficacy against four dermatopathogenic strains: *C. albicans*, *C. glabrata*, *S. aureus*, and *M. luteus*. While the antimicrobial properties of essential oils have been studied previously, this research specifically focuses on dermatopathogenic microorganisms that cause skin infections. This targeted approach provides unique insights into the potential applications of these essential oils in dermatological preparations. The comprehensive evaluation of both bacterial and fungal pathogens and the assessment of enzymatic inhibition properties offers a novel perspective on the dermatoprotective potential of these essential oils.

## 2 Material and Methods

### 2.1 Plant Material

The study focused on two native plant species found in the northeastern Moroccan province of Oujda (34° 41′ 17.0″ N, 1° 54′ 41.0″ W): *T. vulgaris* and *C. nepeta*. The aerial parts of both plants were collected during late spring (May 2024) from local markets in Oujda. The vendor (STE ANGAD NATURE OUJDA, Common Business Identifier: 000031254000042) sourced the plants from local cultivation. Plant materials were air‐dried in the shade at room temperature (22–25°C) for 14 days until constant weight was achieved. The dried plant material was then ground to a coarse powder using a mechanical grinder. Whole plant samples, including leaves, stems, and flowers, were used for taxonomic identification. Voucher specimens (TV‐2024‐011 for *T. vulgaris* and CN‐2024‐015 for *C. nepeta*) were deposited at the Herbarium of the Faculty of Sciences, Mohammed First University in Oujda. Prof. Mohammed ADDI, a botanist at the Faculty of Sciences, performed the taxonomic identification based on morphological characteristics.

### 2.2 Essential Oils Extraction

According to the procedures set by Taibi et al., and detailed in their individual works, the essential oils (EOs) of *T. vulgaris* and *C. nepeta* were extracted by hydrodistillation of the volatile portions [26].

### 2.3 GC‐MS Analysis

Gas chromatography‐mass spectrometry (GC‐MS) was employed to identify and quantify the volatile constituents of the studied EOs. The analysis was performed using a GC Shimadzu system coupled with a MS QP2010. The capillary column used was an RTX‐5 (30 m in length, 0.25 mm internal diameter, and 0.25 µm film thickness) with a coating of 95% dimethyl diphenylpolysiloxane. High‐purity helium (99.99%) served as the carrier gas at a constant flow rate of 1 mL/min. The temperature program was set as follows: the oven temperature was initially maintained at 50°C for 1 min, then programmed to increase at a rate of 3°C per minute until reaching 250°C. The temperatures of the injection port, ion source, and interface were all maintained at 250°C. Sample components were ionized by electron impact (EI) at 70 eV, and the mass analyzer scanned over a range of 40–300 m/z. For sample preparation, 1 µL of EO diluted with an appropriate solvent was injected into the system using split mode with a split ratio of 90:1. To ensure accuracy and reproducibility, three independent analyses were performed for each sample. Compound identification was accomplished by comparing retention times and mass spectra with standards and references available in the NIST library. Internal and external standards were employed for accurate quantification. Data acquisition and analysis were performed using Laboratory Solutions software (v2.5).

### 2.4 Antiaging and Protective Properties

#### 2.4.1 Anti‐Elastase Activity

The anti‐elastase assay was carried out using spectrophotometry in accordance with the protocol that Era et al. outlined with a few minor adjustments. Sterile water was used to dissolve porcine pancreas elastase, yielding a stock solution with a 3.33 mg/mL concentration [27]. Tris‐HCl buffer (pH 8) was used to dissolve the substrate, N‐succinyl‐Ala‐Ala‐Ala‐p‐nitroanilide (AAAPVN), at 1.6 mM. Samples were incubated with the elastase solution in the buffer at room temperature for 15 min, with concentrations ranging from 1 to 300 µg/mL. The synthetic substrate was then added to initiate the reaction. The positive control was oleanolic acid, and the negative control was water. At 400 nm, absorbance was measured using a microplate reader. The proportion of elastase inhibition was calculated using the following formula:

$$\mathrm{Inhibition}\;\;\left(\%\right)=\left[1-\left(\frac{S}{C}\right)\right]\;\times 100$$

where “C” represents the corrected absorbance of the controls (without sample) and “S” represents the corrected absorbance of the test samples. Plotting of dose‐response curves with GraphPad Prism software (San Diego, CA, USA) allowed for determining the IC_50_ value.

#### 2.4.2 Anti‐Tyrosinase Activity

Momtaz et al. described how to measure tyrosinase inhibition activity. L‐3,4‐dihydroxyphenylalanine (L‐DOPA) or L‐tyrosine were the substrates employed in the tests; they were first dissolved in DMSO and then diluted in potassium phosphate buffer (50 mM, pH 6.5) [28]. A Multiskan FC microplate reader was used to take absorbance measurements during the experiments, which were carried out on 96‐well plates. Thirty microliters of tyrosinase (333 units/mL in phosphate buffer, pH 6.5) was mixed with an oil sample (70 µL). The mixture was then incubated for 5 min at room temperature. After that, 110 µL of substrate (2 mM L‐tyrosine or 12 mM L‐DOPA) was added, and the reaction mixture was left to incubate for an additional thirty minutes. Kojic acid was used as the positive control, and a blank contained all components except for L‐tyrosine or L‐DOPA [29]. Absorbance was measured at 492 nm, and the percentage of tyrosinase inhibition was calculated using the following formula:

$$\mathrm{Tyrosinase}\;\mathrm{inhibition}\;\;\left(\%\right)=\left(\frac{A_{\mathrm{control}}-A_{\mathrm{sample}}}{A_{\mathrm{control}}}\right)\;\times 100$$

where A_control_ and A_sample_ stand for the absorbances of the test reaction mixture (which contains kojic acid or oil) and the blank, respectively. The extracts' and kojic acid's IC_50_ values were obtained from linear regression curves and reported in µg/mL.

#### 2.4.3 Sun Protection Ability

According to Mansur et al., the UV absorbance method was used to calculate the sun protection factor (SPF) of APEO [30]. Three measurements at each wavelength were made to determine the absorbance of the EO (0.1 mg/mL) over the wavelength range of 290–320 nm in 5 nm increments. Equation (1) was used to determine the SPF, and Equation (2) was used to determine the percentage of UVB that was blocked:

(1) $$\mathrm{SPF}\;=\;\mathrm{CF}\times \sum_{290}^{320}\;\left[EE\left(\lambda \right)\times I\left(\lambda \right)\times Abs\;\left(\lambda \right)\right]$$

(2) $$\%\;\mathrm{Blocked}\;\mathrm{UVB}\;=\left[1-\left(\frac{1}{SPF}\right)\right]\;\times 100$$

The erythemogenic impact of light at wavelength λ is represented by *EE*(*λ*), the spectrophotometric absorbance at wavelength *λ* is represented by *Abs*(*λ*), the solar intensity spectrum is represented by *I*(*λ*), and the correction factor (10) is represented by CF. The constant values for *EE*(*λ*) × *I*(*λ*), as determined by Sayre et al. [31], are shown in Table 1.

**TABLE 1:** Relationship between erythemogenic effect and radiation intensity.

Wavelength (nm)	*EE* × *I* (Normalized)
290	0.0150
295	0.0817
300	0.2874
305	0.3278
310	0.1864
315	0.0837
320	0.0180
Total	1

### 2.5 Antimicrobial Activity

#### 2.5.1 Inoculum Preparation

Bacterial and fungal strains were obtained from the Microbial Biotechnology Laboratory, Faculty of Sciences, Oujda, Morocco. Bacterial strains included Gram‐positive *S. aureus* (ATCC 6538) and *M. luteus* (LB 14110). Yeast strains included *C. albicans* and *C. glabrata*.

Bacterial strains were cultured on Mueller Hinton agar (MHA) for 18 h at 37°C, while yeast strains were grown on potato dextrose agar (PDA) for 48 h at 25°C. For both microorganism types, cell suspensions were prepared by adjusting the concentration to 10^6^ cells/mL using optical density measurements at 600 nm with a UV‐visible spectrophotometer [32, 33].

#### 2.5.2 Disc Diffusion Method

The activity was determined using the disc diffusion method. The procedure involves inoculating Petri dishes containing MHA/PDA medium with a standardized concentration of microbial suspension. Subsequently, the discs of filter paper (Whatman No. 3) with 6 mm of diameter were placed on the medium surface. Then, 15 µL of samples were impregnated. The positive controls were cycloheximide (1 mg/mL) for yeast strains and gentamicin (1 mg/mL) for bacterial strains. The resulting inhibition diameters around the wells were measured, and all assays were carried out in triplicate [34].

#### 2.5.3 Minimum Inhibitory Concentration (MIC), Minimum Bactericidal Concentration (MBC), and Minimum Fungicidal Concentration (MFC)

To determine the MIC, the methods outlined by Taibi et al. [26]. A total of 0.15% agar was used as the diluent to dilute the EH. As previously mentioned, the MIC test was conducted with a concentration range of 16%–0.0078%, utilizing the same 96‐well microplate technique. The approximate concentration of the microbial suspension utilized in the experiment was 10^6^ cells/mL. The microplates were incubated at 37°C for 18 h for bacterial strains and at 25°C for 24 h for yeast strains. 20 µL of resazurin was added to each well after the incubation time to make it easier to detect growth.

A 3 µL aliquot from each well was added to the MHA and incubated for 24 h at 37°C to calculate the MBC. A 3 µL aliquot from every well was added to the PDA for the MFC, and it was incubated for 48 h at 25°C. After the incubation time, it was discovered that the MBC and MFC were the lowest EO concentrations at which there was no discernible microbial growth.

### 2.6 Statistical Analysis

All experiments were performed in triplicate, and results are expressed as mean ± standard deviation (SD). Statistical analysis was performed using GraphPad Prism software (version 9.0, San Diego, CA, USA). One‐way analysis of variance (ANOVA) followed by Tukey's post‐hoc test was used to determine significant differences between EOs and standard drugs. Differences were considered statistically significant at *p* < 0.05.

## 3 Results and Discussion

### 3.1 Phytochemical Composition

**FIGURE 1:**
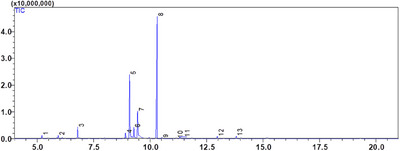
GC/MS chromatogram of the chemical composition of CNEO.

**FIGURE 2:**
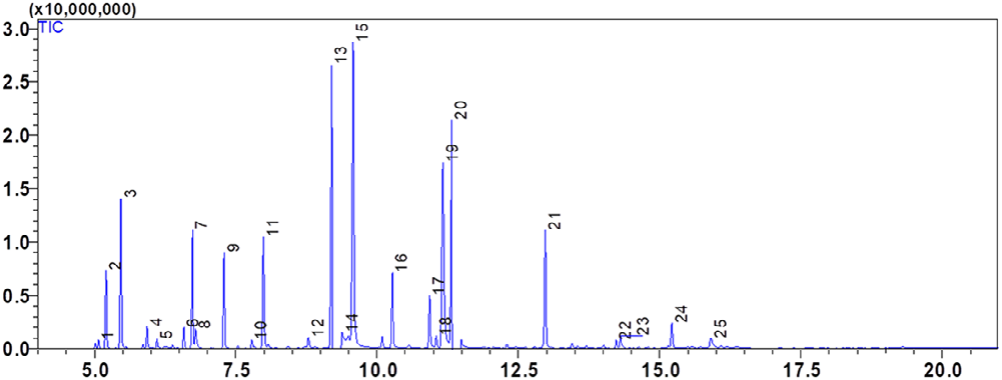
GC/MS chromatogram of the chemical composition TVEO.

The EOs of two Lamiaceae plants, *C. nepeta* and *T. vulgaris*, was examined using gas chromatography and GC‐MS mass spectrometry. The extraction yield was 0.42 ± 0.07% (v/w) for *T. vulgaris* and 1.17 ± 0.05% (v/w) for *C. nepeta*. According to the analysis, 13 compounds were found in the CNEO and 25 in the TVEO (Table 2; Figures 1 and 2).

**TABLE 2:** Chemical composition of *C. nepeta* and *T. vulgaris* essential oils.

				Area (%)	
N°		Compounds	RT	*C. nepeta*	*T. vulgaris*
1	Monoterpenes	α‐Thujene	5.075	—	0.35
2		α‐Pinene	5.205	1.15	3.41
3		Camphene	5.467	—	6.52
4		β‐Pinene	5.932	1.06	1.04
5		β‐Myrcene	6.106	—	0.51
6		β‐Cymene	6.581	—	0.97
7		α‐Terpinene	6.732	—	5.01
8		D‐Limonene	6.790	3.54	1.51
9		γ‐Terpinene	7.289	—	4.32
10		4‐Carene	7.785	—	0.51
11	Oxygenated monoterpenes	β‐Linalool	7.990	—	5.32
12		Camphor	8.788	—	0.78
13		Menthone	8.908	2.05	—
14		Dihydrocarvone	9.091	23.25	—
15		Borneol	9.197	—	9.39
16		Isomenthone	9.274	4.35	—
17		Isoborneol	9.386	—	1.39
18		Neoisomenthol	9.443	17.21	—
19		α‐Terpineol	9.577	—	19.82
20		Thymoquinone	10.271	—	4.16
21		Pulegone	10.305	42.33	—
22		3‐Carvomenthenone	10.522	0.68	—
23		2‐Camphanol acetate	10.930	—	2.83
24		β‐Isosafrole	11.045	—	0.85
25		Carvacrol	11.164	0.73	13.47
26		*O*‐Cymen‐5‐ol	11.315	1.28	6.38
27	Sesquiterpenes	Caryophyllene	12.975	1.28	7.26
28		Germacrene D	13.810	1.09	—
29		γ‐Muurolene	14.233	—	0.36
30		δ‐Cadinene	14.307	—	0.91
31	Oxygenated sesquiterpenes	Caryophyllene oxide	15.214	—	1.65
32		τ‐Cadinol	15.905	—	1.28

The results reveal that CNEO consists mainly of oxygenated monoterpenes, representing 91.88% of the compounds detected, with pulegone as the major component (42.33%). In contrast, TVEO has a more balanced composition, with 24.15% monoterpenes, 64.39% oxygenated monoterpenes, 8.53% sesquiterpenes, and 2.93% oxygenated sesquiterpenes. The dominant compounds in TVEO are α‐terpineol (19.82%) and carvacrol (13.47%).

Common compounds identified in EOs include α‐pinene, β‐pinene, D‐limonene, o‐cymen‐5‐ol, and caryophyllene, each with potential implications for dermatoprotection [35]. For example, β‐pinene, known for its anti‐inflammatory and skin‐soothing properties [36], is present at 1.15% in *C. nepeta* and 3.41% in *T. vulgaris*, suggesting that *T. vulgaris* may offer better protection against skin inflammation.

When comparing these results with previous studies, such as those by Akbar [37] and Kazemi [38] on *T. vulgaris*, where a strong presence of carvacrol was observed, our results show a similarity in terms of oxygenated monoterpenes, but with variations in specific percentages. Similarly, research by Vlachou et al. on wild adult plants of *C. nepeta*, growing in Oropos, Attica (lat. 38° 17′ 28.5″ N, long. 23° 50′ 43.5″ E), also indicated a predominance of oxygenated monoterpenes, which corresponds to our results although the percentages differ slightly [22]. Another study, such as that of Benkhaira et al., showed that the essential oil from the aerial part of the Sefrou region (Middle Atlas of Morocco: 33° 49′ 50″ N, 4° 50′ 15″ W) identified 76.79% of oxygenated monoterpenes, followed by monoterpene hydrocarbons (18.65%) and sesquiterpene hydrocarbons (2.76%). The main constituents were 1,8‐cineole (22.80%), piperitone oxide (14.78%), and limonene (10.73%) [39]. This difference in chemical composition may explain the variations in biological properties and therapeutic applications of the essential oils of these two plants.

### 3.2 Antiaging and Protective Properties

Evaluation of the anti‐elastase activity of CNEO and TVEO revealed promising results. CNEO demonstrated more potent inhibition of porcine pancreatic elastase, with an IC_50_ of 13.55 ± 0.81 µg/mL, compared with TVEO, which had an IC_50_ of 28.40 ± 2.64 µg/mL (Table 3). Although these values are higher than those of oleanolic acid (IC_50_ = 8.41 ± 0.67 µg/mL) used as a positive control, they nevertheless indicate significant potential for antiaging applications. The difference in efficacy between CNEO and TVEO could be attributed to the specific composition of bioactive compounds in each EO, suggesting that CNEO constituents may have a higher affinity for the elastase active site.

**TABLE 3:** IC_50_ of essential oils on elastase, monophenolase, and diphenolase activities.

EO/control	Mushroom tyrosinase activity IC_50_ (µg/mL)		Elastase
	Monophenolase activity	Diphenolase activity	
CNEO	36.71 ± 4.09^b^	22.77 ± 0.97^a^	13.55 ± 0.81^b^
TVEO	47.50 ± 2.68^c^	30.26 ± 3.56^b^	28.40 ± 2.64^c^
Kojic acid	16.96 ± 1.04^a^	36.78 ± 2.62^c^	—
Oleanolic acid	—	—	8.41 ± 0.67^a^

*Note*: Values represent mean ± SD (*n* = 3). Different superscript letters within the same column indicate significant differences (*p* < 0.05) according to one‐way ANOVA followed by Tukey's post‐hoc test.

Both EOs demonstrated inhibitory activity against fungal tyrosinase, for both monophenolase and diphenolase activity (Table 3). CNEO showed greater inhibitory activity than TVEO in both cases, with IC_50_s of 36.71 ± 4.09 µg/mL and 22.77 ± 0.97 µg/mL for monophenolase and diphenolase activities, respectively, compared with 47.50 ± 2.68 µg/mL and 30.26 ± 3.56 µg/mL for TVEO. Interestingly, both EOs showed better inhibition of diphenolase activity than monophenolase activity. This observation could be explained by a preferential interaction of the active compounds with the intermediates of the enzymatic reaction rather than with the initial substrate.

Compared with kojic acid (positive control), the EOs showed comparable or superior activity for inhibition of diphenolase activity, suggesting promising potential for depigmenting applications. These results are in line with previous studies demonstrating the anti‐tyrosinase properties of EOs from the Lamiaceae family [40].

Evaluation of the sun protection capacity revealed interesting results for both EOs (Table 4). CNEO demonstrated a calculated SPF of 6.472, blocking 84.55% of UVB, while TVEO presented an SPF of 5.640, blocking 82.27% of UVB. Although these values are lower than those of commonly used broad‐spectrum synthetic sunscreens, they nevertheless indicate a significant sun protection capacity.

**TABLE 4:** Spectrophotometric absorbances of hydroalcoholic aliquots of TVEO and CNEO, along with the percentage of UVB inhibited and estimated SPF.

Wavelength (nm)	*EE*(*λ*) × *I*(*λ*)^**^ Employed	Absorbance^*^	
		CNEO	TVEO
290	0.0150	0.5870 ± 0.0278	0.6817 ± 0.0061
295	0.0817	0.5933 ± 0.0114	0.6087 ± 0.0090
300	0.2874	0.5763 ± 0.0090	0.5687 ± 0.0097
305	0.3278	0.6573 ± 0.1196	0.5240 ± 0.1718
310	0.1864	0.7587 ± 0.0462	0.5920 ± 0.1103
315	0.0837	0.7230 ± 0.0359	0.6003 ± 0.0511
320	0.0180	0.5277 ± 0.0260	0.4577 ± 0.0229
Calculated SPF		6.472	5.640
% UVB blocked^***^		84.55%	82.27%

* Values represent the mean absorbance values ± standard deviation from three measurements, *n* = 3.

** Constant values of the erythemogenic effect (*EE*) of radiation at wavelength *λ* multiplied by the solar intensity (*I*) at wavelength *λ* as determined by Sayre et al. [31].

*** Percentage of UVB blocked was calculated using the formula in Section 2.4.3.

The existence of chemicals with UV absorption capabilities may be the cause of the performance disparity between CNEO and TVEO. These findings align with earlier research demonstrating the sun‐protective qualities of some EOs [41].

Notably, the elastase and antityrosinase activity observed can be attributed to the richness of EO in bioactive terpene compounds. Studies have shown that oils rich in pulegone, a major compound in CNEO, possess remarkable antityrosinase and anti‐elastase activities, as demonstrated by the study carried out by Cheraif, K. et al. [42]. Other studies have also revealed that carvacrol, which is one of the main constituents of TVEO, could play a key role in skin protection. In particular, Games et al. have demonstrated the anti‐elastase efficacy of carvacrol [43]. In addition, Jeon et al. also demonstrated the antitityrosinase efficacy of carvacrol [44].

The remarkable results obtained for TVEO are in line with other studies notably that carried out by Mhiri et al., which demonstrated that TVEO from Tunisia possesses powerful anti‐elastase and anti‐tyrosinase properties, suggesting its potential as an ingredient in cosmetic and pharmaceutical preparations [45]. This consistency between our results and those of previous studies reinforces the validity of our observations and underlines the growing interest in these EOs in the field of dermatoprotection. Their anti‐elastase, anti‐tyrosinase, and sun protection properties make them promising candidates for the development of natural antiaging, depigmenting, and photoprotective cosmetics.

### 3.3 Antimicrobial Activity Against Dermatopathogenic Micro‐Organisms

Evaluation of the antimicrobial activity of CNEO and TVEO against four dermatopathogenic germs revealed promising results, highlighting their therapeutic potential in the field of dermatology (Table 5).

**TABLE 5:** Antimicrobial activity of CNEO and TVEO.

		*M. luteus*	*S. aureus*	*C. glabrata*	*C. albicans*
CNEO	15 µL^*^ of Essential oil, IZ^**^	35.60 ± 0.65^b^	≥60^a^	≥60^a^	31.13 ± 0.60^b^
	MIC (% v/v)	2.00 ± 0.00	0.50 ± 0.00	0.50 ± 0.00	0.75 ± 0.35
	MBC (% v/v)	4	2	2	4
TVEO	15 µL of Essential oil, IZ	40.83 ± 0.35^a^	42.13 ± 0.25^b^	44.03 ± 0.30^b^	45.86 ± 0.28^a^
	MIC (% v/v)	0.046 ± 0.02	0.093 ± 0.04	0.011 ± 0.00	0.011 ± 0.00
	MFC (% v/v)	0.25	0.25	0.125	0.0625
	15 µL Gentamicine (1 mg/mL) IZ (mm)	26.86 ± 0.25^c^	27.16 ± 0.45^c^	—	—
	15 µL Cycloheximide (1 mg/mL) IZ (mm)	—	—	21.50 ± 0.20^c^	23.70 ± 0.36^c^

*Note*: All values in this table represent the mean ± SD (*n* = 3). Different superscript letters within the same column for each parameter indicate significant differences (*p* < 0.05) according to one‐way ANOVA followed by Tukey's post‐hoc test.

* Used volume for Disc Diffusion Method.

** Diameter of inhibition zone (mm).

CNEO demonstrated significant antimicrobial activity, particularly against *S. aureus* and *C. glabrata*, with zones of inhibition ≥60 mm. This marked efficacy against these two pathogens suggests an interesting potential for the treatment of associated bacterial and fungal skin infections. However, its activity was relatively less pronounced against *M. luteus* (35.60 ± 0.65 mm) and *C. albicans* (31.13 ± 0.60 mm), indicating a certain selectivity in its antimicrobial action.

TVEO, on the other hand, exhibited remarkable and more uniform antimicrobial activity against all the pathogens tested. Inhibition zones ranged from 40.83 ± 0.35 mm for *M. luteus* to 45.86 ± 0.28 mm for *C. albicans*. This widespread efficacy suggests a broad spectrum of action, which is particularly advantageous in the context of polymicrobial skin infections.

A notable aspect of this study is the MIC values obtained. TVEO showed remarkably low MIC values, ranging from 0.011 ± 0.00% v/v for *Candida* spp. to 0.093 ± 0.04% v/v for *S. aureus*. These values are significantly lower than those of CNEO (0.50%–2.00% v/v), indicating a superior antimicrobial potency of TVEO. Interestingly, both EOs showed particular efficacy against *Candida* strains. Given the increasing prevalence of cutaneous fungal infections and the challenges associated with their treatment, this observation is of considerable clinical importance.

Comparison with reference antibiotics (Gentamicin and Cycloheximide) highlights the potential efficacy of these EOs as natural alternatives. In some cases, notably for *T. vulgaris*, the zones of inhibition observed were greater than those of conventional antibiotics, suggesting a promising therapeutic potential. Because both EOs are rich in bioactive substances, including terpenes and phenolic compounds, their antibacterial activity has shown outstanding results. Pulegone, the main component of CNEO oil, has been shown by Farhanghi et al. to be effective against *S. aureus* [46]. Another study by Boni et al. demonstrated the antifungal efficacy of pulegone on a wide range of *Candida* strains [47].

Several studies have also highlighted the antimicrobial efficacy of carvacrol, the majority compound in TVEO. The study by Memar et al. and that by Mączka et al. demonstrated the antimicrobial efficacy of this compound against a wide range of pathogenic strains [48]. These results corroborate and explain the observations made in our study, underlining the therapeutic potential of these EOs in the treatment of skin infections.

These results confirm other observations, such as those reported by Öztürk, G et al., who showed that this essential oil native to Turkey exhibited notable antimicrobial activity against a wide range of microbial strains [49]. Other studies concur with the remarkable results obtained for *T. vulgaris*, such as those reported by Borugă et al., who showed that the TVEO tested possessed strong antimicrobial properties [50]. These results corroborate and explain the observations made in our study, highlighting the therapeutic potential of these essential oils in the treatment of skin infections.

This study highlights the significant potential of CNEO and TVEO as natural antimicrobial agents against dermatopathogenic germs. Their efficacy against a wide range of pathogens, coupled with their low MIC, positions them as promising candidates for the development of new therapeutic strategies in dermatology.

## 4 Conclusion

This comparative study of CNEO and TVEO has highlighted their significant potential in dermatoprotection and antifungal applications. Our results show that these essential oils possess remarkable anti‐elastase, anti‐tyrosinase, photoprotective, and antifungal properties, opening up new prospects for their use in cosmetic and pharmaceutical formulations. CNEO, studied for the first time in this context, stood out for its superior efficacy in most tests. Its unique chemical composition, dominated by oxygenated monoterpenes and particularly pulegone, seems to be behind these impressive biological properties. These innovative results position CNEO as a promising candidate for the development of natural dermatoprotective products. TVEO, although slightly less effective than CNEO in our tests, confirmed its potential already recognized in the scientific literature. Its more diversified chemical composition contributes to a broad spectrum of biological activities, reinforcing its interest for varied applications in dermatology and mycology. The antifungal activities observed against *C. albicans* and *C. glabrata* underline the potential of these essential oils in the fight against cutaneous fungal infections, a field of growing importance in dermatology.

## Ethics Statement

No ethical approval or consent was needed for this study, as the experimental work does not involve animal or human subjects.

## Conflicts of Interest

The authors declare no conflicts of interest.

## Data Availability

The data that support the findings of this study are available from the corresponding author upon reasonable request.
